# G-Quadruplexes and the DNA/RNA helicase DHX36 in health, disease, and aging

**DOI:** 10.18632/aging.203738

**Published:** 2021-12-04

**Authors:** Aaron Antcliff, Louise D. McCullough, Andrey S. Tsvetkov

**Affiliations:** 1 Department of Neurology, The University of Texas McGovern Medical School, Houston, TX 77030, USA; 2 The University of Texas Graduate School of Biomedical Sciences, Houston, TX 77030, USA; 3 UTHealth Consortium on Aging, The University of Texas McGovern Medical School, Houston, TX 77030, USA

**Keywords:** DHX36, RHAU, G4R1, G-quadruplex DNA, G-quadruplex RNA

## Abstract

G-Quadruplex (G4) DNA (G4 DNA) and RNA (G4 RNA) are secondary nucleic acid structures that have multiple roles in vital cellular processes. G4 DNA- and RNA-binding proteins and unwinding helicases associate with and regulate G4s during virtually all processes that involve DNA and RNA. DEAH-Box helicase 36 (DHX36), a member of the large DExD/H box helicase family, enzymatically unwinds both G4 DNA and G4 RNA. By exerting its G4 helicase function, DHX36 regulates transcription, genomic stability, telomere maintenance, translation and RNA metabolism. This review will provide an overview of G4s and DHX36, including DHX36’s potential role in neuronal development and neurodegeneration. We conclude with a discussion of the possible functions of G4s and DHX36 in the aging brain.

## INTRODUCTION

Guanine-rich nucleic acid structures called G-quadruplexes (G4) found in DNA and RNA motifs have become a focal point for the investigation of how secondary structures in nucleic acids affect cellular processes [1, 2]. As non-canonical nucleic acid structures, G4s have unique roles in gene expression, replication, recombination, translation, and telomere maintenance activity [1, 2]. G4s have been extensively investigated in cancer cells, and some anti-cancer therapeutic agents specifically target G4 DNA [3]. Recently, research has turned to brain cells to understand how G4s regulate neuronal aging and neurodegeneration [4–7].

DHX36 (also known as RHAU and G4R1), a highly conserved member of the DExD/H box helicase family, binds with and unfolds G4s, thereby changing how G4 structures influence DNA- and RNA-dependent processes. DHX36, a major G4 helicase, unwinds both G4 DNA and G4 RNA, and understanding the impact on both structures makes it an interesting research target, considering the role of G4s in a variety of processes [8]. Recent reviews on the subject extensively described DHX36’s structure and how DHX36 functions in cells [9, 10]. In this minireview, we provide a brief overview of G4/DHX36 interaction and a brief summary of recent discoveries in the field with emphasis given to DHX36’s role in cancer, telomere maintenance, and viral infection. We then discuss the role of DHX36 in neurodegenerative diseases and brain aging and highlight possible future directions for research in these areas.

## G4 DNA

**Figure 1:**
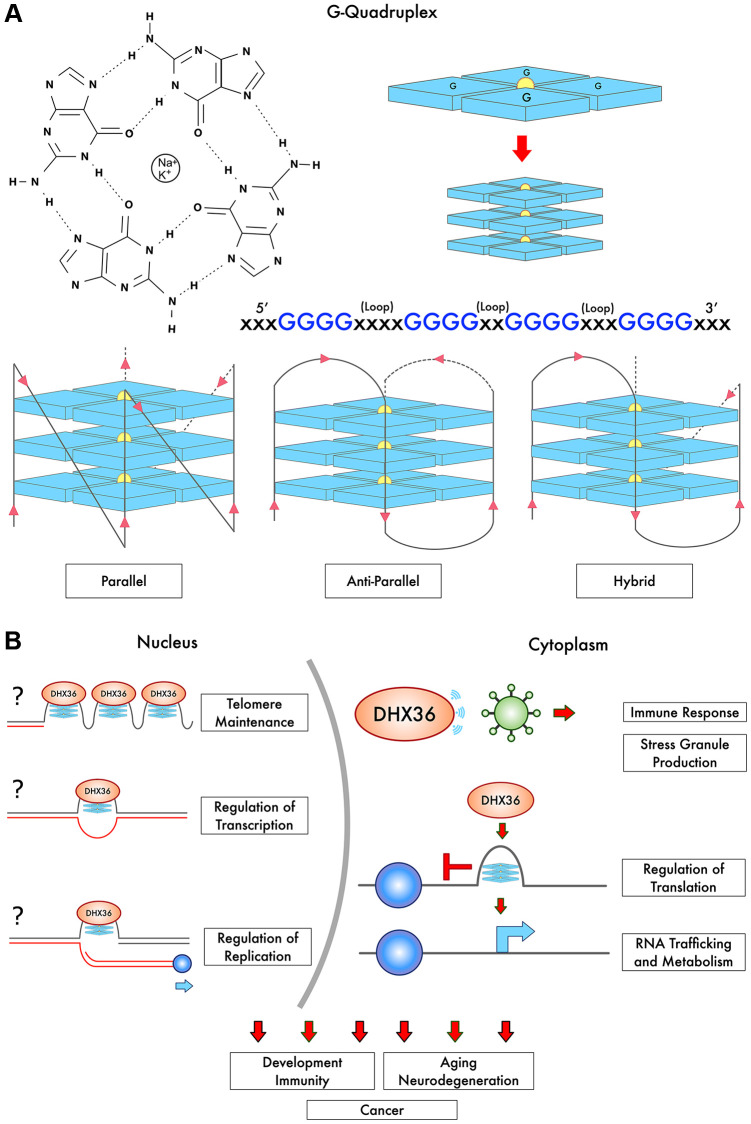
**G-Quadruplex structure, strand arrangement, and interactions with DHX36.** (**A**) Tracts of guanines form planar arrangements via Hoogsten-bonding into G-quartets (blue) and are stabilized by a cation bonded to oxygen molecules in the center (Na^+^ or K^+^). Multiple G-quartets then stack to form G-quadruplexes (G4), in varying strand arrangements. (**B**) DHX36 regulates telomere maintenance, translation including non-AUG translation and RNA trafficking. DHX36 appears to regulate transcription and likely modulates replication (depicted with the question mark). The question marks also indicate that the direct binding of DHX36 to telomeric G4s and G4s in gene promoters *in vivo* remains to be shown. DHX36 is also involved in the innate immune response. Thus, multiple biological processes are influenced, including development, immunity, tumorigenesis, aging, and neurodegeneration.

Canonical Watson-Crick base pairing features pairs of matched nitrogenous purine and pyrimidine hydrogen-bonded bases, stably structured on anti-parallel phosphate-sugar strands. Guanine nucleosides form non-canonical secondary structures via electronic self-association. G4 structures are composed of guanine-rich tracts of DNA, which have bonded together via Hoogsten base pairing in cyclical planar arrangements called G-quartets (Figure 1A). G-quartet-forming tracts contain sequences of at least four guanine bases in the following representative sequence: >G_>X_>G_>X_>G_>X_>G_>X_, wherein each guanine (>G) is interspaced by a linking nucleotide sequence (_>X_) [1]. The quartets are linked via loops formed by the nucleotides between each G-run, and these strands may run in parallel, anti-parallel, and hybrid configurations [1]. Multiple adjoining G-quartets can stack into elongated three-dimensional tetrads, which are stabilized via a central shared monovalent cation, most often K+ or Na+, to form stable G4 DNA structures. G4 stability also depends on loop length and composition, and is affected by sequences of the loops connecting G-runs [1]. Stability is influenced by chromosomal location, such as telomere vs. promoter regions, and by diversity of nucleotides within the tract [1].

G4 DNA is densely represented in function-specific genomic areas, such as promoters, promoter enhancement regions, oncogenes, and to a high degree, telomeres, all pointing to specific and important functional roles of the G4 structures [1, 2, 11]. Telomeres, G-rich single-stranded 3′ extensions at chromosome ends (TTAGGG) in the form of overhangs, are the most common region in which G4s form. G4 DNA is localized to many nucleosome-depleted gene promoters, effectively acting as a stabilizing platform, in which transcription can begin [12–14]. Indeed, a large fraction (approaching 50%) of human genes contain G4 motifs within their promotor regions, highlighting a central role in regulation of gene expression in terms of transcriptional and inhibitory functions. For example, G4s act as high-affinity docking and binding sites for transcription factors, leading to expression of many genes [12–14]. Numerous transcription factors, including SP2, NRF1, FUS, MYC, YY1, and ZHX1, interact with G4 structures in the human genome [14]. Conversely, G4s also have roles in inhibiting transcription. Intra- and intermolecular G4 DNA formations act as physical structural barriers on both template and non-template strands, creating conditions in which the RNA polymerase complex is blocked from transcribing DNA templates [12], creating loss of function. Thus, G4 DNA structures have extensive activator and repressor roles that modulate a variety of vital processes in cells.

## G4 RNA

Due to their innate single-stranded nature and intramolecular preference for parallel-stranded conformations, G4 RNAs are more thermally stable than G4 DNA structures, as confirmed in *in vitro* conditions [2]. Like G4 DNA, RNA-based G-quadruplex structures play significant roles in a variety of molecular processes. Most important are mRNA-related functions of translation, splicing, polyadenylation, and termination [2]. In stressed cancerous cells, mature cytoplasmic transfer RNAs (tRNA) are cleaved to produce tRNA fragments, tRNA-derived stress-induced RNAs (tiRNAs), which assemble into intermolecular G4 RNA and function to repress translation [15]. Recently, studies featuring the stabilization of G4 RNA via pyridostatin-based drugs described how translation efficiency is affected. For example, in stem cells, specifically targeting G4 RNA with a small-molecule G4 stabilizer, carboxypyridostatin, results in the production of oligodendrocyte progenitors [16]. The stabilizer, a derivative of pyridostatin, stabilizes a G4-forming sequence in the SARS-CoV-2 genome in live cells and reduces translation, thus lowering protein levels of COVID-19 [17]. More research is required to understand G4 RNA functions in coronaviruses.

## A major G4 DNA and RNA helicase, DHX36

Helicases are specialized enzymes driven by ATP hydrolysis that unwind DNA and RNA and function in transcription, replication, genomic stability, telomere maintenance, virus identification, and immune response mechanisms [9]. An estimated 1% of genomes in both eukaryotic and prokaryotic life encode for helicases that are classified into super-families [9]. Since G4 DNA and G4 RNA are structurally heterogeneous, there are many G4 helicases that can resolve various G4s. The most investigated mammalian G4 helicases are PIF1 (petite integration frequency 1), BLM (Bloom syndrome protein), WRN (Werner syndrome protein), RTEL1 (regulator of telomere elongation helicase 1), FANCJ (Fanconi anemia group J helicase), and DHX36 (DEAH box protein 36) [1].

Both G4 DNA and RNA can be unwound by a member of the DExH Box resolvase enzyme family (in which x can be any amino acid), DHX36, which has high specificity for G4 structures^5^. Intriguingly, DHX36 was originally described as an RNA-associating protein that binds to the AU-rich elements in the 3′ region of mRNAs and enhances mRNA degradation [18]. Co-crystallization of DHX36 bound to G4 DNA demonstrated that the N-terminal DHX36 motif folds into a DNA-binding-induced alpha-helix that specifically binds parallel G4 DNA structures [8]. The helicase is ubiquitously expressed in humans and mice.

DHX36 knockout is embryonically lethal, showing that DHX36 is vitally important (Figure 1B) [19]. DHX36 conditional knockdown in the hematopoietic system causes hemolytic anemia, reduced proliferation and cell-cycle defects, ineffective differentiation of progenitors and stem cells, and deregulation of genes containing a G4 DNA motif in their promoters [19]. Eukaryotic mRNAs contain a modified guanine at their 5′ ends that serves as a “cap”, and recruitment of mRNA to ribosomes occurs by cap-dependent and cap-independent mechanisms, and DHX36 plays a role in both processes [20]. Recently, DHX36 was shown to play a role in C9orf72 repeat-associated non-AUG translation in amyotrophic lateral sclerosis [21]. The activity of G4 helicases regulated by phosphorylation or acetylation [22], but it is still not clear how DHX36 is modulated. The functions of DHX36 in cellular homeostasis have been extensively studied, particularly in the areas of cancer, telomere maintenance, and antiviral immune response.

## DHX36 in cancer

**Figure 2:**
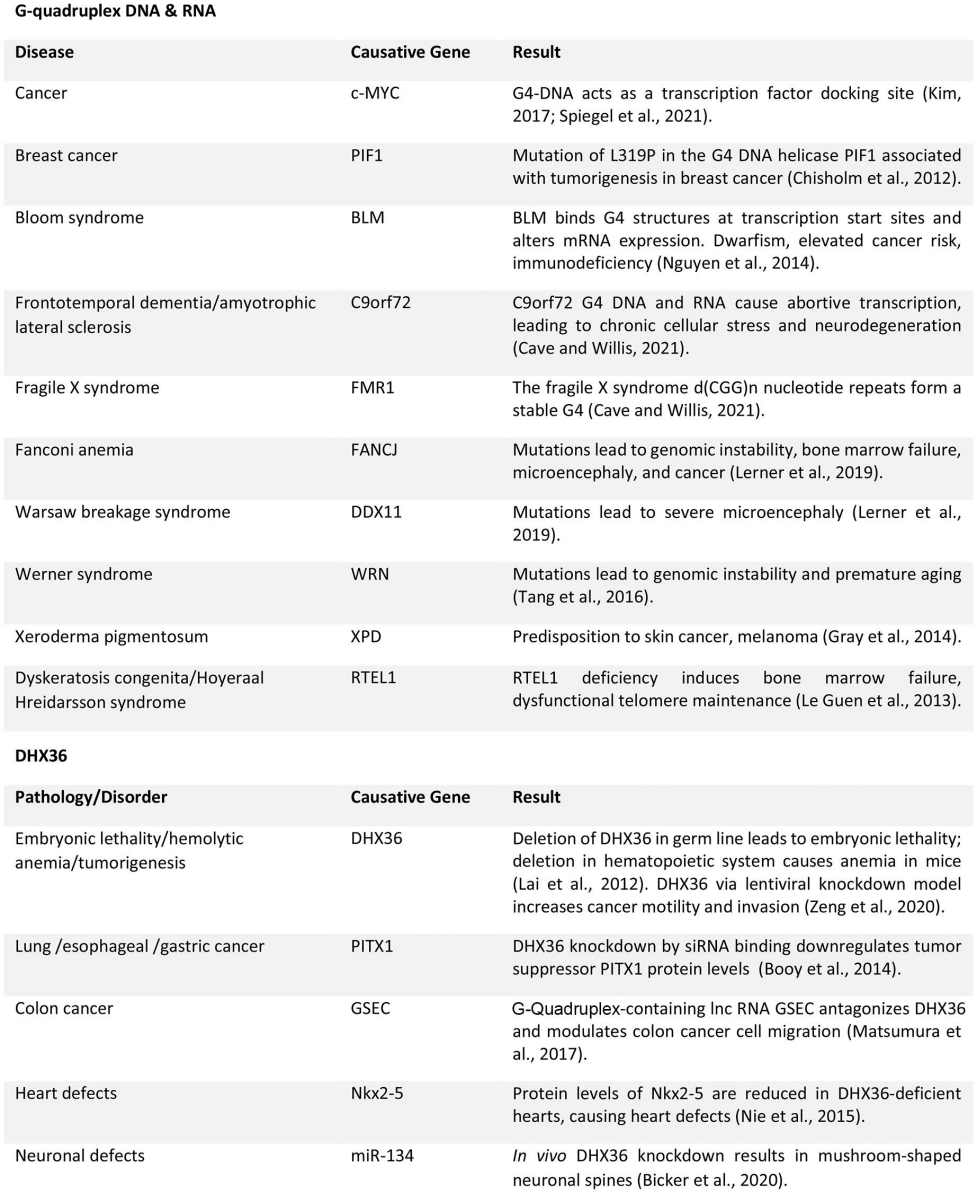
Summary of G-quadruplex-associated dysfunction in various conditions [44–50].

Due to the ubiquity of G4 structures in promoters, promoter-enhancement regions, and oncogenes, much of helicase study focuses on how these enzymes unwind DNA and RNA and their impact on cancer tumorigenesis [3]. Helicase enzyme levels are increased in several cancers and mutations or deletion of helicase genes often lead to cancer (Figure 2) [23].

DHX36 is a proto-oncogene. The long non-coding RNA, FLJ39051, that contains G4 RNA, binds to and inhibits DHX36, leading to enhanced colon cancer cell migration [24]. Since some proto-oncogenes contain G4 motifs within promotor regions (e.g., c-MYC [14]), DHX36 activity may regulate their expression and, thereby, modulate cell proliferation. DHX36 regulates pre-mRNA 3′-end processing by specifically unwinding parallel G4 RNA to maintain p53 pre-mRNA 3′-end processing and, therefore, to orchestrate DNA damage responses [25].

Nevertheless, DHX36 is implicated as a regulating factor in breast cancer tumor suppression, and is positively associated with increased patient survival. Using a xenograft tumor model in mice, researchers showed that lentiviral knockdown of DHX36 increases tumor growth and cancer cell invasion, with a concurrent reduction of cellular apoptosis [26]. Therefore, at least in some cancers, DHX36 may function as a tumor suppressor [26]. Overall, DHX36 plays substantial roles in some forms of cancer, with multiple mechanisms related to tumorigenesis.

## DHX36 and the telomerase RNA component

Telomeres have a central role in aging by protecting the ends of chromosomes from damage during cellular mitosis [1]. *In vivo* and *in vitro*, DHX36 binds to the telomerase RNA component, and this binding depends on the presence of the G4 RNA structure in the 5′-region of the telomerase RNA [27]. DHX36 resolves G4 RNA in the RNA component of telomerase and promotes the formation of a stem-loop structure, which is important for reverse transcription by telomerase [28]. Telomere extension is prevented by small-molecule G4 ligands, leading to cell senescence and further supporting the involvement of the G4 structures in telomere maintenance [29]. In agreement with these findings, siRNA-mediated downregulation of DHX36 results in lowered telomerase function and reduced telomeres [30]. Intriguingly, in cancerous cells, the telomerase RNA component can be exported to the cytoplasm and translated, generating the telomerase RNA protein (TERP) that functions in cell stress pathways [31]. It would be interesting to determine if the G4 RNA structure within the telomerase RNA and DHX36 regulates production of TERP. Thus, more research is needed to understand DHX36-dependent molecular mechanisms of telomerase regulation.

## DHX36 in viral infection

Helicase enzymes, including DHX36, have an active role in innate cellular immune response, acting as cytoplasmic sensors of viral nucleic acids [32]. Among other helicases, DHX36 regulates antiviral responses in myeloid dendritic cells. During viral infections, the helicase activity of DHX36 induces PKR, which results in SG formation and leads to initiation of immune responses [33]. In experiments in a *dhx36* tamoxifen-induced knock-out cellular system, DHX36-deficient cells displayed deficits in IFN production and had higher vulnerability to RNA virus infections [33]. Thus, DHX36 is important in cellular defense mechanisms via augmenting the production of cytoplasmic antiviral factors.

Viruses, such as coronaviruses, hijack the host machinery for their replication, and helicases are no exception. Coronaviruses encode their own RNA helicases, but they still hijack the host helicases to regulate virus proliferation. For example, the host helicases, including DHX36, may in fact positively modulate SARS-CoV-2 replication [34].

## A potential role of DHX36 in neurological and neurodegenerative diseases

Many G4 DNA helicases are linked to human diseases with some degree of brain pathology, often severe. For example, an expansion of a non-coding GGGGCC repeat in the gene C9ORF72 is associated with 10% of familial cases of frontotemporal dementia and amyotrophic lateral sclerosis. The repeat forms G4 DNA, G4 RNA, and G4 DNA-RNA hybrids, leading to an accumulation of ribonucleoprotein complexes and increased intranuclear stress [35]. G4s are implicated in fragile X syndrome caused by trinucleotide CGG expansion and silencing of the fragile X mental retardation gene [35]. Fanconi anemia, in which the FANCJ G4 helicase is mutated, leads to genomic instability, bone marrow failure, microcephaly, and cancer [36]. In Warsaw breakage syndrome, a mutation in the G4 DNA helicase DDX11 causes severe microcephaly [36]. Dyskeratosis congenita, a bone-marrow-failure syndrome, is linked to mutations in telomeric G4 helicase RTEL1 and leads to microcephaly, developmental delay, and intracranial calcifications [36] (Figure 2).

DHX36 knock-out causes lethality in embryos that show signs of cellular degeneration at the early embryonic stage E7.5 [19]. Is there any indirect evidence that DHX36 is involved in neurological or neurodegenerative diseases? Intriguingly, in cultured hippocampal neurons, DHX36 controls dendritic localization of precursor-miRNA-134 (pre-miR-134), and as a result, it modulates spine morphogenesis, a process regulated by miR-134 [37]. Cultured neurons with DHX36 downregulated by shRNA exhibit more mushroom-shaped spines with increased spine volume, compared to control neurons, indicating that DHX36 is involved in synaptic plasticity [37]. shRNA-mediated Dhx36 knockdown inhibits BDNF-induced dendritic morphogenesis [38]. Lastly, in neurons, the 5′UTR of Task3 mRNA, an mRNA of an acid-sensitive potassium channel, folds into a parallel G4 structure, and DHX36 appears to regulate dendritic localization and expression of Task3, further suggesting that the helicase is important for neuronal physiology [39]. Thus, dysfunctional DHX36 may lead to neurological and neurodegenerative diseases, which remains to be elucidated.

## DHX36 and the aging brain

As described above, many G4 DNA helicases are linked to human diseases, which are characterized by accelerated aging. Cellular senescent phenotypes arise from genomic instability, telomere dysfunction, inflammation, dysregulated transcription, translation, and mRNA metabolism that are the result of the presence of defective or absent helicases. For example, in knockdown assays, the closely related helicase DHX9 that unwinds G4 DNA, G4 RNA, and R-loops causes premature senescence in human fibroblasts. These cells have significant morphological differences, changing cell shape from a small and spindle shape to an enlarged and irregularly shaped appearance. Positive staining of the widely used senescent marker SA β-gal was also observed when compared to controls [40].

As humans age, declines occur in the systems for controlling protein production quality and mitochondrial function and dynamic processes regulating cellular homeostasis. Stress granule formation, maintenance and clearance processes may become compromised and lead to promotion of aging-related disease and dysfunction. For example, G4s have long been recognized as factors in stress granule assembly [41]. Intriguingly, in specific neuronal populations, motor neurons in particular, G4 RNA in tRNA-derived, tiRNA trigger a neuroprotective response in a translational repressor Y-box binding protein 1 (YB-1)-dependent manner [42].

As a multifunctional helicase, DHX36 may participate in controlling aging and cellular senescence processes in neurons. Previously, we showed that G4 DNA is important in regulating autophagy, an essential cellular survival and maintenance process [4, 6]. We found that pharmacologically stabilizing G4 DNA reduces *Atg7*expression in neurons and astrocytes, suggesting a mechanism by which G4 DNA downregulates autophagy. Another example is G4-associated transcription of the *Brca1* gene [5]. In neurons, stabilizing G4 DNA with small-molecule G4 stabilizers leads to *Brca1* downregulation and DNA damage [5]. Intriguingly, stabilizing G4 DNA fails to downregulate *Brca1* in astrocytes and microglia, indicating differences in DNA damage and repair pathways between brain cell types [7]. Given its well-defined interactions with G4 DNA, DHX36 may act as a regulator of autophagic gene expression and DNA damage responses, influencing cellular senescence for many cell types within the aging brain. Further research is needed to understand how DHX36 and related helicases can be used for understanding the mechanisms of brain aging.

## Conclusions and future directions

Although the interaction of DHX36 and G4 structures has been characterized across a variety of biological processes, much work is still needed to understand the mechanistic roles controlled by this dynamic relationship. As reviewed here, G4 DNA, G4 RNA, and DHX36 together define behavior for many genes at transcriptional and post-transcriptional levels. DHX36 may be an important genome instability hotspot worth deeper investigation in age-associated diseases. Though much attention has been given to the G4 structures and DHX36 interactivity in cancer tumorigenesis [43] and viral pathology mechanisms [33], important questions have yet to be answered about their role in neurodegenerative diseases and aging. Our previous work showed that G4s are a viable research target in the study of cellular senescence in the aging brain, but more attention should be given to the mechanistic processes occurring in individual cell types. Deeper understanding of the interactions between G4 DNA, G4 RNA and DHX36 within cells may result in the identification of viable treatment options for aging and age-related neurodegeneration.
